# Tracking hidden organic carbon in rocks using chemometrics and hyperspectral imaging

**DOI:** 10.1038/s41598-018-20890-4

**Published:** 2018-02-05

**Authors:** Céline Pisapia, Frédéric Jamme, Ludovic Duponchel, Bénédicte Ménez

**Affiliations:** 1 IPGP, Sorbonne Paris Cité, Univ Paris Diderot, CNRS, 1 rue Jussieu, 75238 Paris Cedex 5, France; 2 Synchrotron SOLEIL, Campus Paris-Saclay, 91192 Gif sur Yvette, France; 3 LASIR CNRS UMR 8516, Université de Lille, Sciences et Technologies, 59655 Villeneuve d’Ascq Cedex, France

## Abstract

Finding traces of life or organic components of prebiotic interest in the rock record is an appealing goal for numerous fields in Earth and space sciences. However, this is often hampered by the scarceness and highly heterogeneous distribution of organic compounds within rocks. We assess here an innovative analytical strategy combining Synchrotron radiation-based Fourier-Transform Infrared microspectroscopy (S-FTIR) and multivariate analysis techniques to track and characterize organic compounds at the pore level in complex oceanic rocks. S-FTIR hyperspectral images are analysed individually or as multiple image combinations (multiset analysis) using Principal Component Analyses (PCA) and Multivariate Curve Resolution – Alternating Least Squares (MCR-ALS). This approach allows extracting simultaneously pure organic and mineral spectral signatures and determining their spatial distributions and relationships. MCR-ALS analysis provides resolved S-FTIR signatures of 8 pure mineral and organic components showing the close association at a micrometric scale of organic compounds and secondary clays formed during rock alteration and known to catalyse organic synthesis. These results highlights the potential of the serpentinizing oceanic lithosphere to generate and preserve organic compounds of abiotic origin, in favour of the hydrothermal theory for the origin of life.

## Introduction

Spectroscopic techniques and especially Synchrotron-based Fourier-Transform Infrared microspectroscopy (S-FTIR) have been used in geosciences for decades^1–3^. S-FTIR has notably been proven efficient for determining at the micrometer scale the mineral, fluid and organic phases contained in interplanetary dust particles, meteorites and terrestrial material^4–10^. When characterizing samples at micrometer scale, user is tracking phases with a very limited spatial distribution and more or less low concentrations. This is typically the case for organic matter trapped within the tiny pores of a mineralized matrix, notably in natural rock samples susceptible to contain abiotic organic molecules of prebiotic interest or biological remnants of ancient or extant life^11–15^. Being able to investigate the nature and origin of these organic compounds at an appropriate scale is then of high importance considering their putative importance for understanding the origin, evolution and distribution of life on Earth.

Current FTIR mapping mode leads to the acquisition of hyperspectral images whose pixels correspond to individual spectra^1^. Exploiting each spectrum allows embracing the heterogeneity and complexity of natural samples. Typical data treatment of FTIR hyperspectral images performed on biological samples consists in establishing distribution maps of selected band areas characteristic of the functional groups of biomolecules such as Amide I at 1600–1700 cm^−1^ (80% C=O stretch; 10% C-N stretch; 10% N-H bend) and Amide II at 1500–1560 cm^−1^ (60% N-H bending and 40% C-N stretching vibrations) for proteins, or C=O groups of ester (1736 cm^−1^) for lipids^16,17^. But this univariate analytical treatment implies to know *a priori* all the species present in the sample in order to check that the spectral information used is specific of the compounds of interest. Additionally, unanticipated compounds are completely disregarded after data processing. Geobiological samples, including numerous minerals and organic compounds, almost always generate complex spectra with overlaps of vibrational bands that are not easy to fit^2^. As an example, C-O vibration of carbonates^18^ occurring at 2872 and 2980 cm^−1^ for calcite may hamper the detection of aliphatic C-H stretching bands between 2800 and 3000 cm^−1^, as do the phyllosilicate H-O-H bending modes at 1620–1640 cm^−1^ with the amide ones^17–19^. Establishing species distribution maps with the integration of one absorption band or a spectral range of infrared spectra may be misleading or incorrect. An appropriate data analysis approach allowing to properly studying multicomponent spectra is then mandatory.

The combined use of S-FTIR microspectroscopy and multivariate image analysis is a very powerful tool that should lead to the accurate characterization of the global and local composition of a complex sample along with the identification of its components^20–22^. Among these chemometric techniques, Principal Component Analysis (PCA) should allow describing the chemical structure and spatial distribution of components of a natural sample^20^. However, principal components never correspond exactly with spectra of pure compounds due to their mutual orthogonality. Multivariate Curve Resolution - Alternating Least Squares (MCR-ALS) is a complementary approach aiming at extracting, without any *a priori*, the contributions of all pure components present in a complex sample. A matrix of individual pure spectra and corresponding concentration profiles, which best fit experimental data, are simultaneously extracted^23,24^.

Both PCA and MCR-ALS analyses were successfully applied to FTIR data collected on natural samples^25^. However these approaches imply that all the components of the sample present a sufficiently-high variance to be extracted from the bulk signal. Heterogeneities in concentrations and spatial distributions sampled by each image pixel will guide the PCA. Variables having a high variance will tend to overwhelm the first principal component. Conversely, variables that do not change much will be roughly considered as a constant and such a low-variance elements will be omitted during analysis^26^. Highly heterogeneously-distributed species such as impurities, which present very low concentrations at the scale of the bulk sample but can be locally abundant, will induce high contributions to individual spectra but only for localized pixels^20^. When considering the whole map, the pixels representative of these impurities will be scarce, with low statistical weight. In order to properly detect and describe impurities by chemometric analysis of hyperspectral images, it is then mandatory to increase the variance of these low concentration compounds. This can be achieved through multiset analysis by simultaneously combining and analysing several images representative of the global variability of one sample. This leads to a more resolved spectral description of the pure components’ contributions when compared to single image analysis^20,27^.

In this study we assess the relevance of combining S-FTIR with PCA and MCR-ALS on both single and concatenated hyperspectral images to unravel complex mixture of minerals and organic compounds in a natural serpentinite from the mantle-derived oceanic crust. Serpentinization reactions result in the large production of molecular hydrogen on Earth^28,29^. This latter derives from the hydration, by circulating hydrothermal fluids, of Fe(II)-rich minerals forming the mantle rocks (i.e. olivine). This production of hydrogen is acknowledged to be responsible for the formation of abiotic organic compounds through the reduction of CO and CO_2_^14,28,30^. Considering the expected reaction rates, these organic compounds may likely represent tiny amounts in the bulk rock and have a highly heterogenous spatial distribution, being most often clustered within tiny pores^31^. They might then be considered as impurities in these complex rocks, in which minerals constitute the dominant phases. We highlight here the importance of multiset analysis for identifying the chemical nature and spatial distribution of pure components in these complex organo-mineral samples. It provides a new analytical strategy for the search and characterization of organic compounds within a mineralized environment in which close spatial relationships between organic and mineral phases may suggest potential genetic links.

## Results

### Spatial distribution of organic compounds relatively to mineral phases in a serpentinite as established using S-FTIR mapping

**Figure 1:**
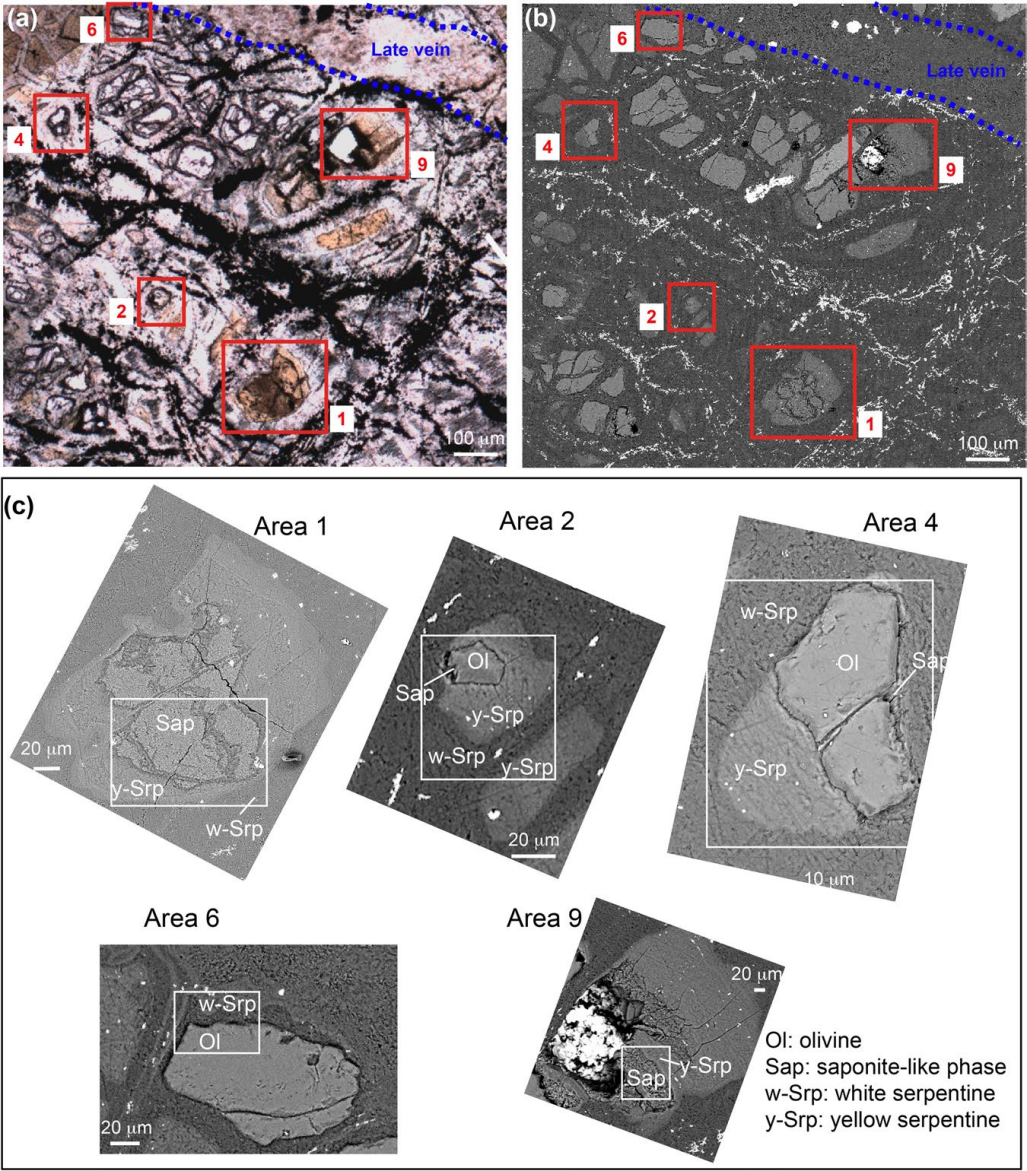
(**a**) Optical microscopy and (**b**) SEM observations of the analysed serpentinized peridotite showing with red rectangles the 5 areas that were considered for PCA and MCR-ALS analysis (numbered Areas 1, 2, 4, 6, 9) along with a late vein crosscutting the sample. (**c**) Enlarged SEM views of the 5 selected areas. White rectangles show locations of S-FTIR hyperspectral images displayed in Fig. 2. The bright zone in Area 9 corresponds to a hole resulting from the removal of an olivine crystal during polishing. SEM images were acquired in backscattered mode between 10 and 15 kV and under high vacuum conditions.

**Figure 2:**
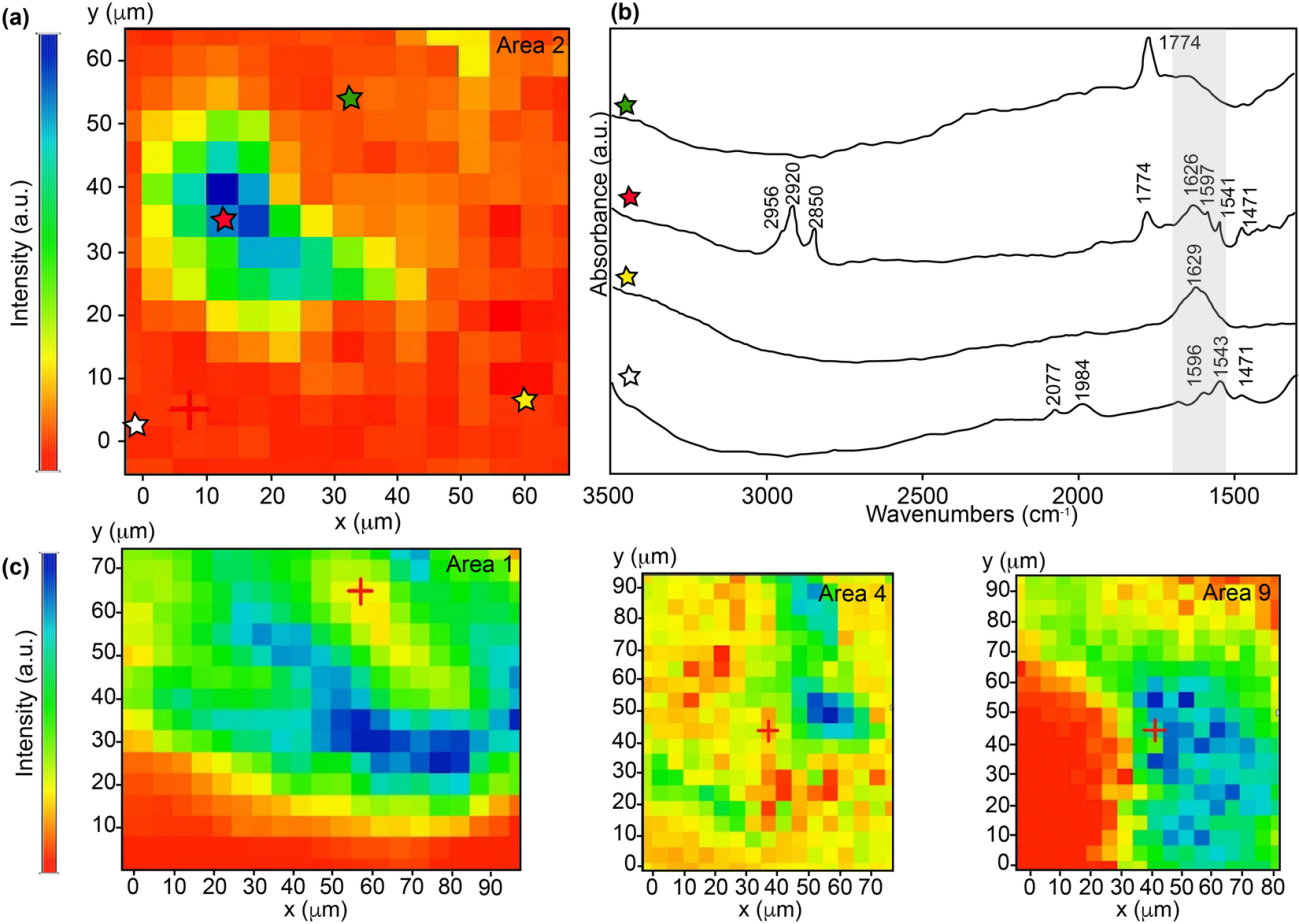
S-FTIR microspectroscopy results with (**a**) the spatial distribution obtained from signal integration at 2848 cm^−1^ considering second derivative spectra and (**b**) examples of raw spectra extracted from four different regions of the map shown in (**a**) and corresponding to olivine (green star), an aliphatic-rich region (red star), white serpentine (white star) and yellow serpentine (yellow star). The light grey rectangle in the 1530–1730 cm^−1^ range illustrates possible overlaps of absorption bands of various mineralogical or organic origin at a given wavenumber (see also Supplementary Table 1 for assignments). **(c)** Spatial distribution obtained from signal integration at 2848 cm^−1^ considering second derivative spectra for Areas 1, 4 and 9 and showing heterogenous distribution of aliphatics throughout the sample (not shown for Area 6 due to the absence of organic signal in this area). a.u. stands for arbitrary unit.

**Figure 3:**
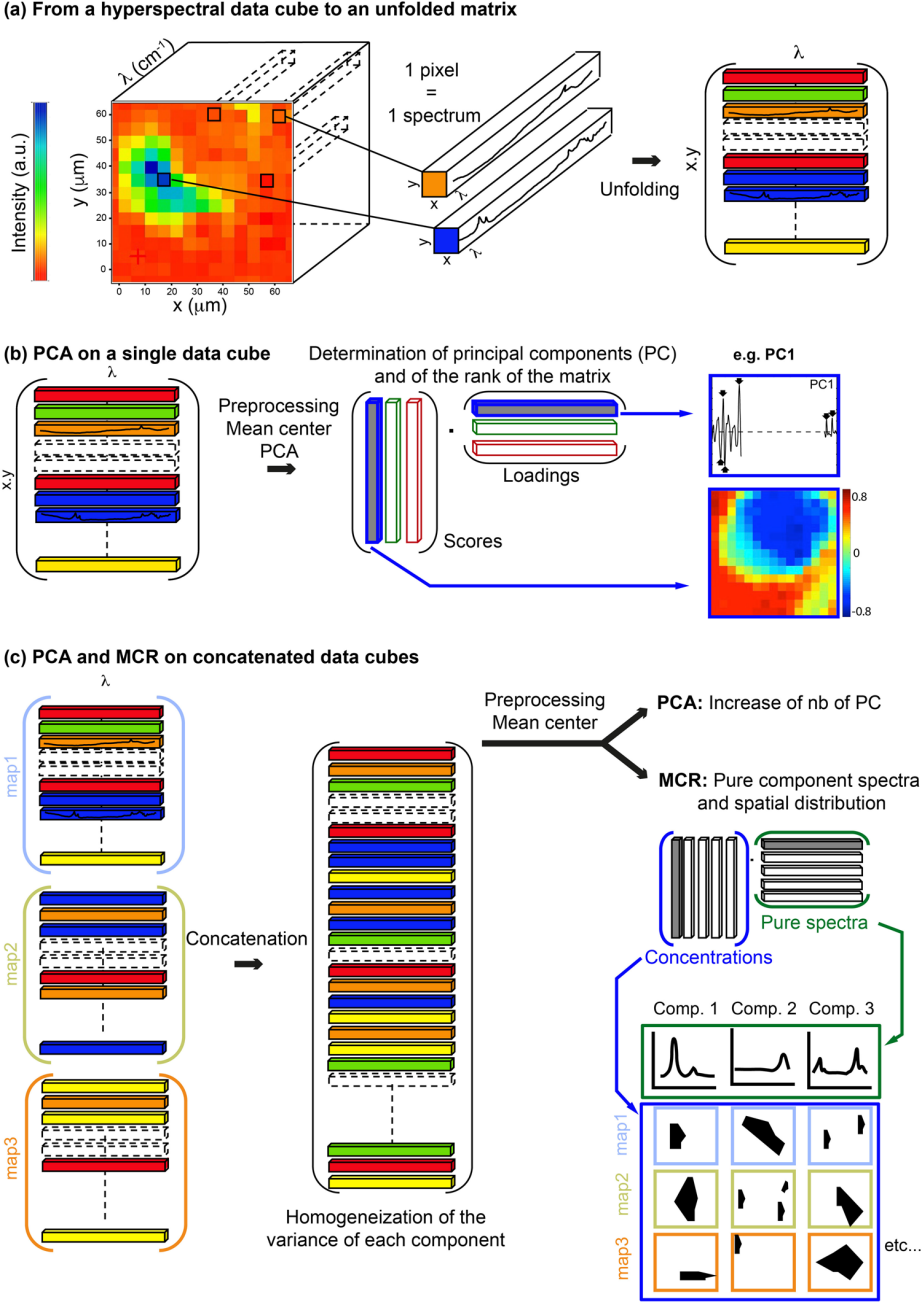
Methodological approach. (**a**) S-FTIR hyperspectral data cubes were first unfolded into a matrix form in which each pixel, which corresponds to an individual spectrum, represents a line of the matrix. (**b**) In order to obtain the scores and loadings of the principal components, PCA analyses were conducted on each individual hyperspectral data cube and (**c**) on concatenated hyperspectral data cubes. MCR-ALS analyses were also performed on concatenated images in order to extract the spectral signature and spatial distribution of the pure components of the system. Before both PCA and MCR-ALS analyses, individual and concatenated images were preprocessed using MATLAB software following the procedure described in Supplementary Figure 6.

An overview of the mineralogical complexity of the sample used in this study and of the five targeted areas are provided in Fig. 1. The mineralogy of the sample was consistent with the post-expedition description of the drilled core (using Raman spectroscopy, SEM-EDX and XRD analyses)^32,33^. The sample exhibited a typical serpentinite pattern (Fig. 1) with a mesh texture made of white serpentine (w-Srp) and opaque iron oxides (i.e. magnetite, Mt), both deriving from the hydration of the primary mantle paragenesis at high temperature. Some olivine kernels (Ol) can nonetheless still be found. Fe-rich yellow serpentines and smectite phyllosilicates (y-Srp and Sap, respectively) present in the core of the mesh texture along with late veining attest for multistage hydration history at lower temperatures during exhumation of the Atlantis Massif^34^. Smectites were identified to be saponite using SEM textural analysis showing characteristic platelets^35^.This is consistent with the mineralogy of the drilled core, saponite being the main secondary clay phase described^32^. Some accessory minerals were also identified as chromium spinels, iron and nickel sulfides and carbonates. The main ranges of vibrational bands observed for the 5 areas of interest and their assignments are given in Supplementary Table 1. An overview of the spatial distribution of organic compounds is given Fig. 2 based on the integration of the CH_2_ stretching band (2848 cm^−1^) intensity. The five areas were representative of the organo-mineral diversity of the sample at a micrometer scale and presented different contents in organic compounds and variable spatial relationships with minerals (Fig. 2a,c). Area 2 presented the most variable mineralogy and a quite strong organic signal (Fig. 2a,b). Optical microscopy, SEM observations, SEM-EDX and S-FTIR analyses indicated the presence of a partly serpentinized olivine rimed with saponite and Fe-rich yellow serpentine, all embedded in a white serpentine matrix (Fig. 1b). Area 4 presented a similar mineralogy to Area 2 (Fig. 1b), with nonetheless less organics (Supplementary Table 1, Fig. 2c). Area 6 was dominated by an olivine mineral embedded into white serpentine (Fig. 1b). No organic signal was detected in this area using S-FTIR microspectroscopy. Finally, Areas 1 and 9 were chosen because they presented a different mineralogical pattern, mainly dominated by Fe-rich yellow serpentine associated with a saponite phase (Fig. 1b). Both images presented a strong organic signal (Fig. 2c). For all areas, most of the S-FTIR spectra were polyphasic. Four typical S-FTIR spectra were identified (Fig. 2b). They presented, respectively: (i) a dominant band at 1774 cm^−1^, which was found to be systematically associated with the presence of olivine (green star), (ii) aliphatics’ vibration bands at 2850, 2920 and 2956 cm^−1^ associated with numerous vibration bands in the 1400–1800 cm^−1^ range that might be attributed either to organic compounds or minerals (red star), (iii) a broad vibration band at 1629 cm^−1^ in the Fe-rich serpentine mineral (yellow star), and (iv) 3 main vibration bands (1472, 1543, 1596 cm^−1^) associated with two weaker ones (1984 and 2077 cm^−1^) in the white serpentine mineral (white star). No evidence for carbonate minerals was found in the five areas analysed in this study. Pure spectral signatures of each component could not be extracted considering that, at a given wavelength, contributions of both minerals and organic compounds could have occurred (Supplementary Table 1 and grey rectangle in Fig. 2b). To achieve this goal, the five selected areas were then analysed using chemometric tools (Fig. 3; see details in Material and Methods section).

### Single and multiset PCA

**Figure 4:**
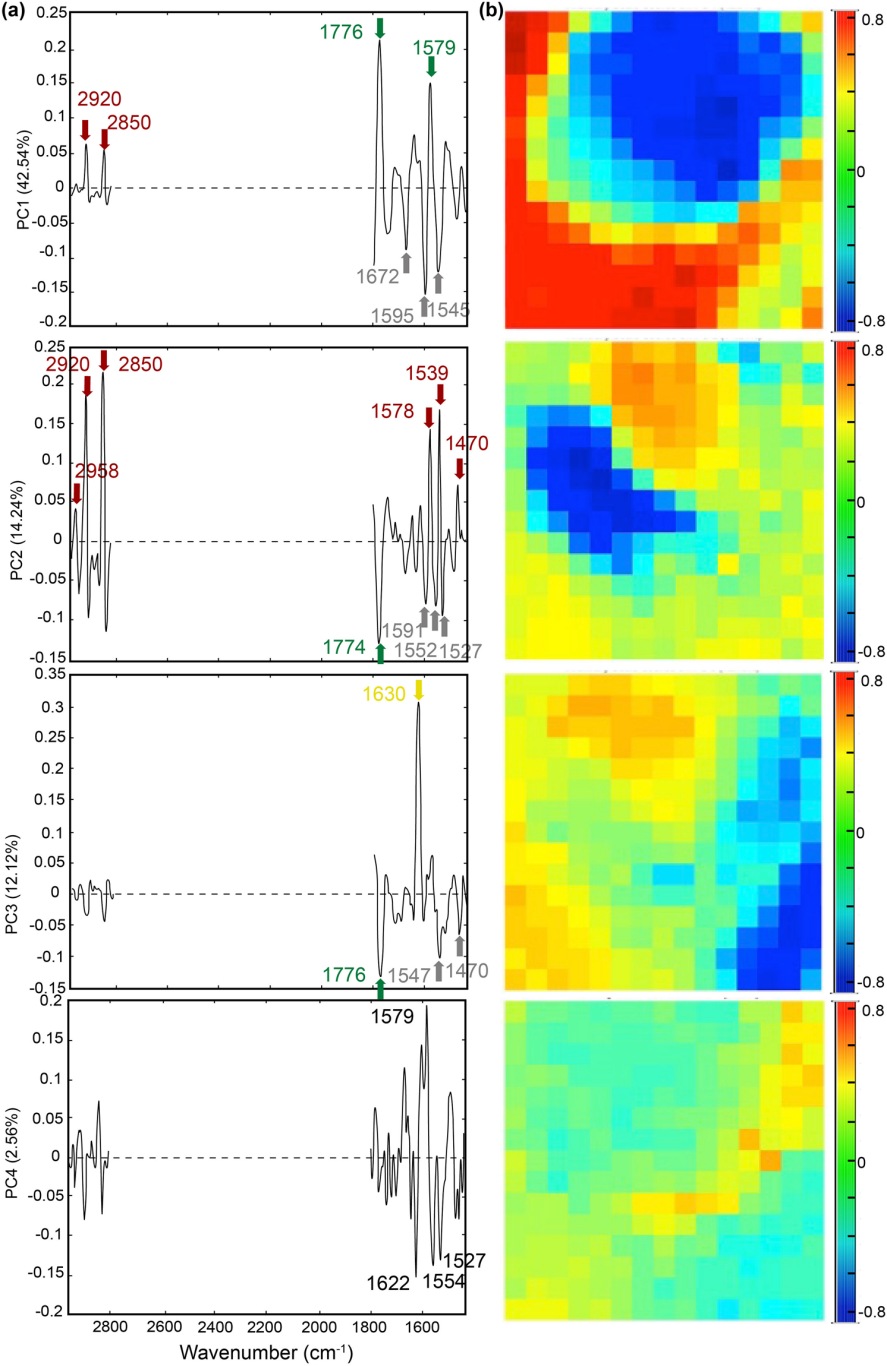
PCA results obtained on individual image Area 2 following preprocessing procedure (i.e. wavenumber range selection, 2^d^ derivative, normalization and mean centering). Loadings (**a**) and scores (**b**) are shown for principal components (PC) 1 to 4. The percentage of total variance explained by the principal component is given along the *y-*axis. Main peaks on loadings are highlighted and were related to organic compounds (red arrows), saponite (yellow arrows), olivine (green arrows) and white serpentine (grey arrows). Peaks values noted in black were not undoubtedly assigned. Note that due to the second derivative process blue colors in score maps correspond to the upper part of the loadings (>0) whereas red colors correspond to the lower part (<0).

After unfolding, the FTIR hyperspectral images were first individually analysed using PCA (Fig. 3a,b). Results obtained on Area 2, which was the most representative of the mineralogical diversity of the sample, are shown Fig. 4. Among the principal components (PC) given by Matlab^®^ software, three were considered as significant (Supplementary Table 2). The first component PC1 corresponded to 42.5% of the total variance (Supplementary Table 2). By comparing scores and loadings (Fig. 4) with phase distributions (Figs 1–2), this component was found to be dominated by the absorption of olivine that was correlated with the main vibrational bands of minor aliphatics but was anti-correlated to white serpentine spectral signature. The second component PC2 explained 14.2% of the total variance and was also polyphasic. It was dominated by peaks characteristic of organic compounds anti-correlated with both white serpentine and olivine vibrational band. The third component PC3 described 12.1% of the total variance. It was dominated by a peak at 1630 cm^−1^ that could be related to the saponite platelets observed after SEM^19^. Interestingly, the associated location of this phase in PC3 scores corresponded to an area that was first assigned to Fe-rich yellow serpentine after optical observations (Fig. 1). This observation may illustrate a possible spatial association of these two minerals. This saponite phase was anti-correlated to white serpentine and olivine, both presenting weak absorption signals. Finally, the fourth component PC4 explained 2.6% of the total expressed variance but the loadings were highly polyphasic and hardly interpretable. The PCA of single Area 2 then clearly showed that the expressed variance was highly dominated by olivine.

These results were consistent with PCA performed on individual Area 4 that presented a similar mineralogical composition compared to Area 2 (Supplementary Figs 1–2). PC1 explained 57.7% of the total variance and was influenced by olivine anti-correlated to white serpentine mineral. Similarly, PC2 was dominated by organic compounds’ vibrational bands jointly with a peak at 1628 cm^−1^ that could be either attributed to amine groups or saponite. PC3 was dominated by clearly-identifiable saponite phase anti-correlated to indistinctive white and yellow serpentines. For all PC, loadings were polyphasic (Supplementary Fig. 2). On the contrary, PCA of individual Area 6, which was also mainly composed of olivine, was not informative. Although only accounting for 37.6% of the total variance, PC1 was the only meaningful PC and allowed differentiating olivine from white serpentine (Supplementary Fig. 2).

Areas 1 and 9 were also individually analysed by PCA (Supplementary Figs 1–2). They presented a similar mineralogy (i.e. Fe-rich yellow serpentine and saponite enclosed in a white serpentine matrix) and were rich in organic compounds. PCA gave similar results even if the number of significant components was higher for Area 1 compared to Area 9 (i.e. 5 against 2). Loadings of principal components were systematically polyphasic (Supplementary Fig. 2). They were mainly dominated by aliphatic stretching bands associated with both saponite and/or Fe-rich yellow serpentine, then highlighting a possible association of organic compounds with these minerals. Yet the variance of the minerals in these areas was not high enough to precisely determine the relationships between all the components of the system.

**Figure 5:**
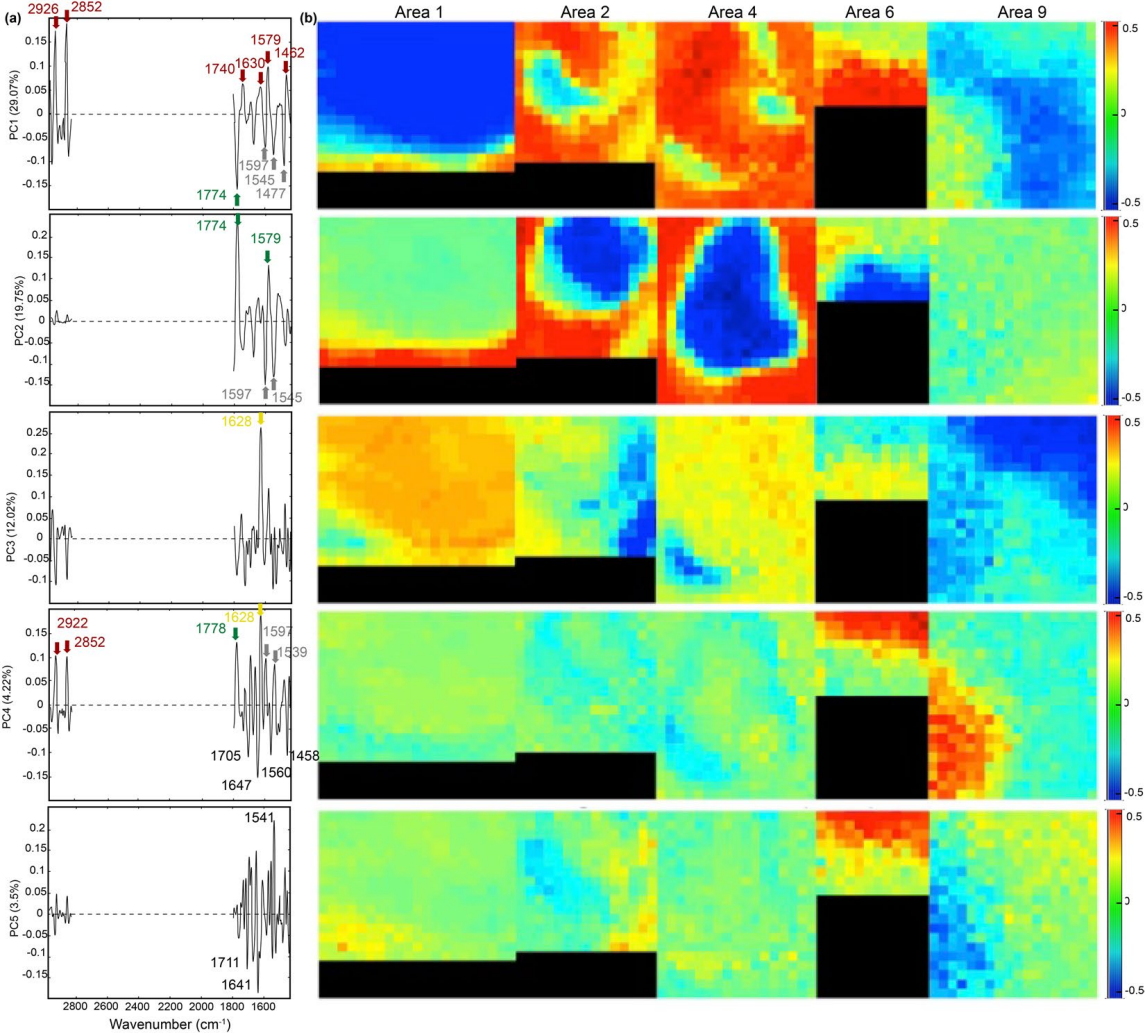
PCA results obtained by combining 5 S-FTIR images (Areas 1, 2, 4, 6 and 9) following the preprocessing procedure (i.e. wavenumber range selection, 2^d^ derivative, normalization and mean centering). Loadings (**a**) and scores (**b**) are shown for principal components (PC) 1 to 5. The percentage of total variance explained by the principal component is given along the *y-*axis. Main peaks on loadings are highlighted and were assigned to organic compounds (red arrows), saponite (yellow arrows), olivine (green arrows) and white serpentine (grey arrows). Peaks values noted in black were not undoubtedly assigned. Note that due to the second derivative process blue colors in score maps correspond to the upper part of the loadings (>0) whereas red colors correspond to the lower part (<0).

Several combinations of data cubes were analysed using PCA in order to test the influence of multiset concatenation on PCs’ extraction (Fig. 3c). Merging two hyperspectral images of similar mineralogy (Areas 2 and 4, Supplementary Table 2 and Supplementary Fig. 3) increased the tendencies observed for PCA of individual data cubes, and failed at separating the organic signal from the mineral ones. The combination of two images exhibiting opposite mineralogical and organic patterns (Areas 2 and 1 or Areas 2 and 9, Supplementary Table 2 and Supplementary Fig. 4) was not relevant either, the expressed variance being totally guided by the dominant phase from Area 1. Full concatenation of all the areas was then analysed by PCA. Results for the five first PCs are given in Fig. 5. The expressed variance was more homogeneously distributed among the different PCs (Supplementary Table 2). By combining loading and score results, several compounds were revealed and minor mineral phases were identified. PC1 (29.1% of the total variance) did not individualize any pure component but was dominated by organic compounds against minerals overwhelming sample composition (white serpentine and olivine). PC2 (19.8% of the total variance) clearly separated two anti-correlated pure phases, namely olivine and white serpentine. PC3 (12.0% of the total variance) only included saponite. PC4 (4.2% of the total variance) was clearly composed of several phases and was less informative. Finally, PC5 (3.6% of the total variance) seemed to individualize a serpentine phase with a distinct spectral signature compared to the ones of the white and yellow serpentines. Even if all the PCs included the contribution of several phases, a higher number of components have been identified after PCA of concatenated data cubes in comparison with PCA of individual images. Indeed, at least parts of the different PC have individualized minerals (e.g. olivine and white serpentine in PC2, saponite in PC3) and organic compounds (PC1). Overall, it was useful to provide a minimum range for the number of pure components to be identified through MCR-ALS analysis.

### Spectral signature and associated spatial distribution of pure components obtained by MCR-ALS analyses

**Figure 6:**
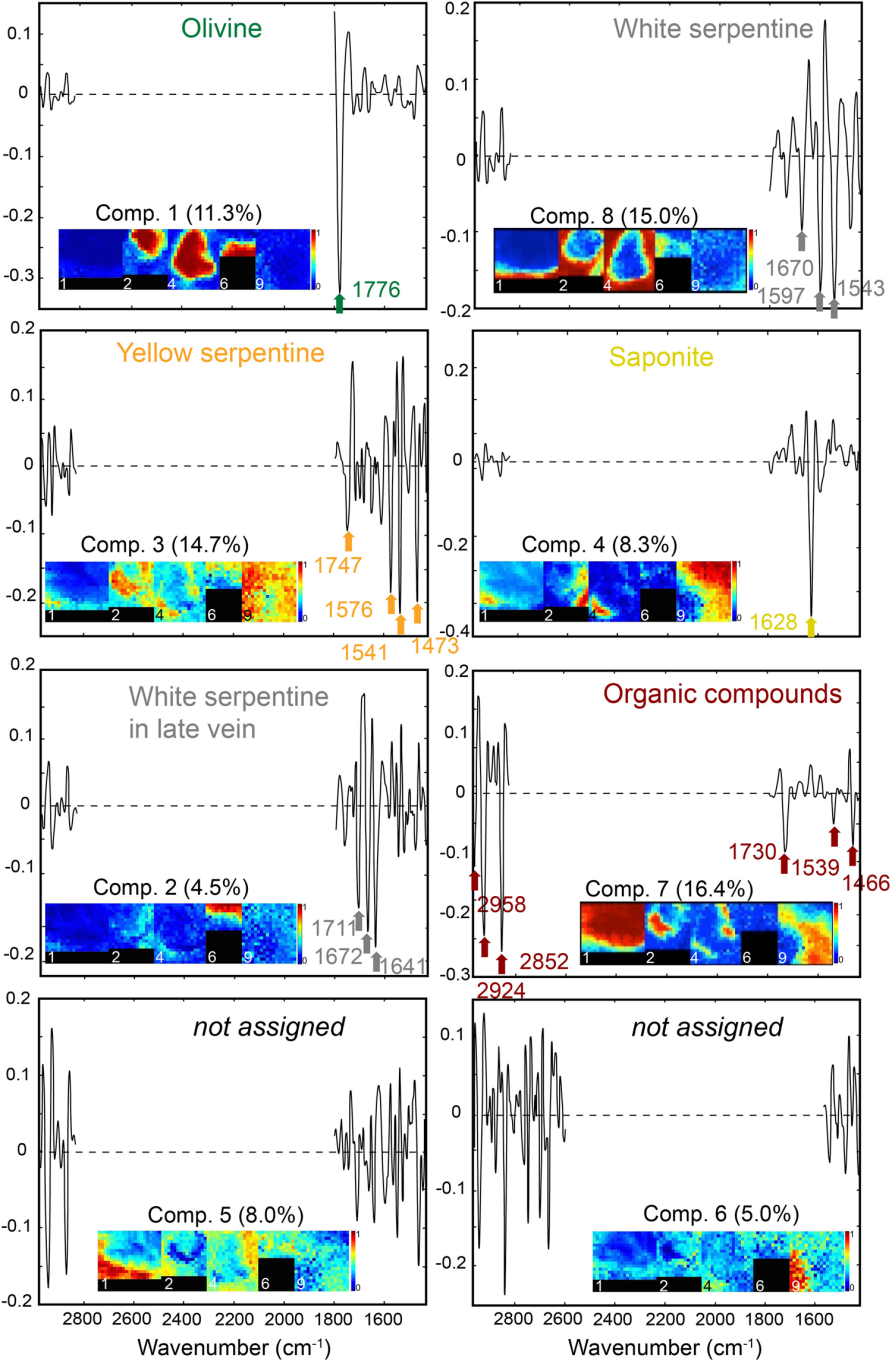
Loadings and scores (as insets) of the 8 pure components (Comp.) identified by MCR-ALS on 5 combined S-FTIR hyperspectral images (Areas 1, 2, 4, 6 and 9) following the preprocessing procedure (i.e. wavenumber range selection, 2^d^ derivative, normalization and mean centering). Six of these pure components (Comp.) were assigned to mineralogical phases or organic compounds (Table 1). Maximum absorbance peaks are mainly visible as negative peaks in loadings.

PCA of hyperspectral data cubes extracts successive orthogonal factors that represent the largest variance of data but do not correspond exactly to spectra of pure compounds. In order to extract the pure chemical spectra and to assess their contributions to the system, MCR-ALS analysis is mandatory. It was performed on concatenated data cubes and 8 pure components were extracted (Fig. 6). Among these 8 components, six can be explained (Table 1) and loadings and scores, which represent the spectral signature and spatial distribution of a pure component respectively, are detailed Fig. 6. One should note that maximum absorbance peaks are mainly visible as negative peaks in the present MCR-ALS loadings. The major minerals of the system corresponded to components 1, 8 and 3. Component 1 was clearly related to the presence of olivine, as confirmed by associated SEM images, and explained 11.3% of the total variance. It was characterized by a marked vibrational peak at 1776 cm^−1^ that may be attributed to Si-O overtone of silicates, although major vibrational peaks of olivine could not be observed due to strong absorption at low wavenumbers. Component 8 was clearly expressed where white serpentine was observed using SEM. It explained 15.0% of the total variance of the system. It presented three main vibrational peaks at 1543, 1597 and 1670 cm^−1^ that could be attributed to the bending of X-O-H (with X = Si, Mg or Fe^36–38^) in phyllosilicates. The third major mineral phase, the Fe-rich yellow serpentine, seemed to be described in component 3, which explained 14.7% of the total variance. It presented three main peaks at 1473, 1541 and 1576 cm^−1^ and a minor peak at 1747 cm^−1^. Interestingly, contrarily to PCA, which was unable to systematically distinguish between amine groups and saponite mineral phase (both having characteristic absorption around 1630 cm^−1^; Supplementary Table 1), MCR-ALS analysis allowed individualizing a component (component 4; 8.3% of the total variance) characterized by a main vibrational peak at 1628 cm^−1^, but without any associated peaks corresponding to organic compounds (e.g. aliphatic moieties). This peak should be attributed to interlayer water of a phyllosilicate lattice^19,39^. In this sample, it should correspond to saponite, which is the main secondary phyllosilicate identified in the drilled core^32^, and consistently with the SEM textural analysis showing at these locations platelets characteristic of this smectite^35^. Similarly, another mineral phase that was not clearly revealed through PCA was represented by component 2 describing 4.5% of the total variance. It was characterized by three main peaks at 1641, 1672 and 1711 cm^−1^. This phase was first identified as white serpentine after SEM observations (Fig. 1) like the one described in component 8. Yet MCR-ALS analyses seemed to indicate that these two white serpentine phases were not identical. This difference may be attributed to the fact they were formed during two distinct hydration phases of the rock, the white serpentine in Component 2 corresponding to a late vein crosscutting the sample, while component-8 related one related to the first high temperature stage of serpentinization. Accordingly, MCR-ALS analysis allowed distinguishing two different types of similarly-looking serpentines. Finally, component 7, which explained 16.4% of the total variance, corresponded to organic compounds. It was characterized by the stretching vibrations of aliphatic CH_2_ and CH_3_ at 2852, 2924 and 2958 cm^−1^ and three main peaks at 1466, 1539 and 1730 cm^−1^ corresponding to aliphatic bending, N-H bending and C=O stretching, respectively. The absence of vibrational band at 1630 cm^−1^ in this pure component confirmed that this peak, which was observed jointly with the organic signal in PCA (Fig. 5), did not correspond to an organic compound but to smectite. The two other components, components 6 and 5, respectively describing 5.0 and 8.0% of the total variance, were not interpretable.

**Table 1:** Details of the main peaks composing the resolved spectra obtained for each pure component using MCR-ALS and corresponding assignments^17,19,36–39,58–60^.

Identification	MCR-ALS components						Band Assignment	References
	1	2	3	4	7	8		
	Olivine	White serpentine in vein	Yellow serpentine	Saponite	Organic compounds	White serpentine		
% variance	11.3	4.5	14.7	8.3	16.4	15.0		
**Peak positions** (cm−^1^)					1466		C-H bending; N-H bending	^17,58,59^
			1473				X-OH bending (X = Si, Mg, Fe)	^36–38^
					1539		N-H bending; C = C stretching	^17,58,59^
			1541			1543	X-OH bending (X = Si, Mg, Fe)	^36–38^
			1576				X-OH bending (X = Si, Mg, Fe)	^36–38^
						1597	X-OH bending (X = Si, Mg, Fe)	^36–38^
				1628			interlayer H-O-H	^19,38,39^
		1641					X-OH bending (X = Si, Mg, Fe)	^36–38^
		1672				1670	X-OH bending (X = Si, Mg, Fe)	^36–38^
		1711					X-OH bending (X = Si, Mg, Fe)	^36–38^
					1730		aromatic C = O stretching	^17,58,59^
			1747				X-OH bending (X = Si, Mg, Fe)	^36–38^
	1776						Si-O secondary order overtone	^60^
					2852		C-H stretching	^17,58,59^
					2924		C-H stretching	^17,58,59^
					2958		C-H stretching	^17,58,59^

**Figure 7:**
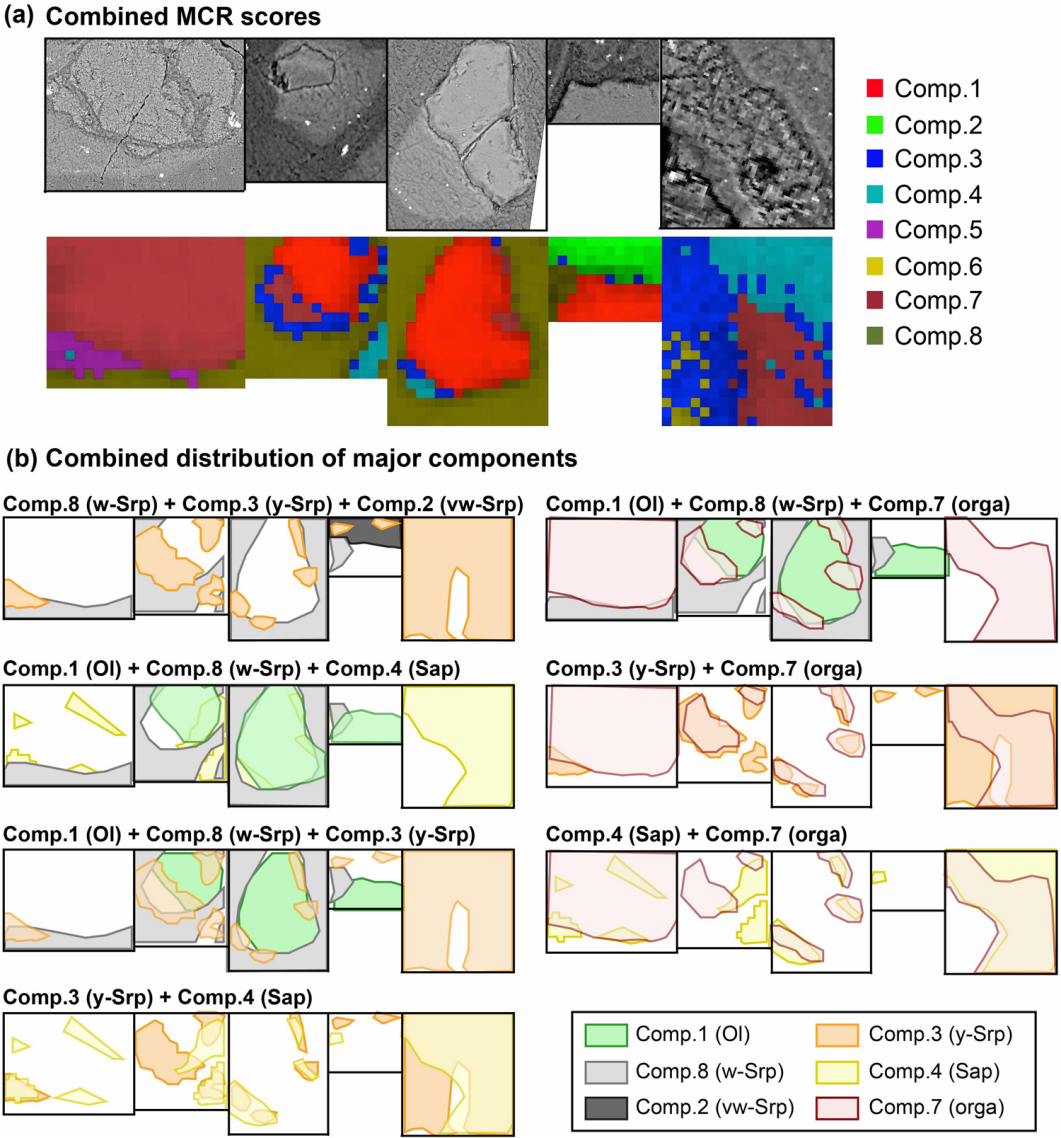
(**a**) Combined MCR-ALS scores. SEM observations of the corresponding Areas are given for comparison. (**b**) Schematic representations of some overlaps of the spatial distribution of the six components (Comp.) of interest among the 8 pure components identified by MCR-ALS. It highlights that organic compounds are mainly spatially associated with saponite and Fe-rich yellow serpentine minerals. Ol, w-Srp, vw-Srp, y-Srp, Sap, orga stand for olivine, white serpentine, white serpentine in late vein, yellow serpentine, saponite and organic compounds, respectively.

By looking at the relative spatial distributions of the identified components (Fig. 7), we determined the relationships between mineral phases and organic compounds. The combined distribution of mineral phases indicated that (i) olivine and white serpentine were well individualized, (ii) yellow Fe-rich serpentine and saponite are intimately spatially associated, and (iii) yellow Fe-rich serpentine and saponite are located at the interface between olivine and white serpentine. Consequently, these two mineral phases seem to indicate reactive interfaces during mineral transformations occurring during serpentinization reactions. Finally, the distribution of organic compounds clearly showed that they are spatially linked to both yellow serpentine and saponite minerals, hence also associated with reactive interfaces.

## Discussion

The results obtained in this study indicate that chemometrics applied to S-FTIR imaging has a great potential for elucidating the chemical heterogeneity of complex geo(bio)logical samples. Principal component analyses performed on individual S-FTIR hyperspectral images were strongly influenced by the dominant phase of the imaged area, either of mineral (for Areas 2 and 4) or organic (for Areas 1 and 9) origin (Fig. 4 and Supplementary Figs 1–2). The percentage of expressed variance described by the first PC was quite high and the other principal components were not informative enough to decipher the complexity of the sample and to establish neither the spectral signature of individual components nor their relative spatial distribution. The use of a multiset approach clearly improved the characterization of the components of the sample, of both organic and mineralogical origin (Fig. 5 and Supplementary Fig. 5). But the multiset matrix has to be carefully designed. Combining two images with a similar pattern reinforced the influence of the dominant phase and was not efficient neither for extracting the organic signal from the mineral one, nor for determining the spatial relationships between the different phases (Supplementary Fig. 5 and Supplementary Table 2). To efficiently achieve these goals, PCA requires a sufficiently-high number of pixels for each component and the associated spectra need to be sufficiently distinct from one phase to another. The accuracy of the resolved spectra will strongly depend on the existence of map pixels or sample spectra reflecting only the contribution of one single chemical component. When transposing this idea to natural geobiological samples, it means that the user has to analyse and combine a sufficient number of samples/areas presenting distinct mineral and organic contents. The total percentage of expressed variance explained by all the significant principal components drops but a higher number of pure components is identified (Supplementary Fig. 5). Although counter-intuitive, it implies that, when tracking impurities such as organic compounds in a mineralized matrix, the user has to focus not only on organic-rich areas but also on organic-poor areas. Targeting distinct areas of the sample, each of them being representative of different organo-mineral associations, would then limit the influence of the high variability of such samples at a micrometer scale and would enable characterizing low-content compounds such as organic compounds in multi-mineral rock.

Chemometrics applied to multiset data was efficient at characterizing the pure components of the system and at precisely distinguishing mineral phases that were not accurately identified by optical and SEM observations (i.e. the two generations of white serpentine) or by S-FTIR univariate analysis. These results can be linked to our knowledge of the history of the sample. They help to discuss the mineralogical transformations induced by successive aqueous alteration phases that occurred during the exhumation of the ultramafic rocks forming the Atlantis Massif^32,34,40^. White serpentine was formed from olivine hydration at high temperature during early hydrothermal circulations while Fe-rich yellow serpentine was assumed to form later, at a lower temperature and a shallow depth^41^. Both presented a similar spectral signature (Table 1) but with slight differences concerning the band positions characteristic of the bending of hydroxyl groups associated with structural cations. The enrichment in iron of the yellow serpentine relatively to the white serpentine might explain the different S-FTIR spectral signatures of these two serpentine phases. Similarly, the spectral signature of the MCR-ALS pure component 2 (vw-Srp) differed from the one of the mesh-forming white serpentine (w-Srp; i.e. component 8), although they display similar aspects. As attested by the presence of the vein (Fig. 1a), this latter phase was affected by late aqueous fluid circulation that may have induced mineral dissolution and elemental remobilization, thus potentially modifying its spectral signature.

One major achievement of the chemometric approach concerns its capability to establish the spatial distribution of the different phases and their relationships. Notably, the spatial distribution of the saponite phase was hardly resolved alone using optical microscopy or S-FTIR univariate analyses. On the contrary, MCR-ALS scores precisely showed a close spatial association between saponite and yellow serpentine pure components (Fig. 7b). Saponite is commonly regarded as a product of late aqueous alteration of serpentinized ultramafic rocks, including in the Atlantis Massif, at temperatures below 150 °C^32,34^. MCR-ALS analyses then seemed to confirm that saponite could have had a genetic link with Fe-rich yellow serpentine and may have been formed during late alteration stages. Similarly, chemometrics was more efficient than S-FTIR univariate analysis at determining the relationships between these mineral phases and the nature and spatial distribution of organic compounds of the sample (Figs 6–7). When comparing the spatial distribution of the organic compounds inferred from the 2848 cm^−1^ peak intensity (Fig. 2a,c) with the scores of the principal components dominated by these organic compounds (PC2 in Fig. 4 and PC1 in Fig. 5), one can notice significant differences, which are enhanced when looking at the distribution of the corresponding MCR-ALS pure components (Figs 6,7). Establishing species distribution from one selected peak in S-FTIR data leaded to a reduced spatial distribution of organics mainly highlighting areas with high local concentrations of these impurities but nonetheless no specific relationship with mineral locations. On the contrary, the definition of pure components using MCR-ALS clearly showed that saponite, whose spatial distribution was intimately linked to the Fe-rich yellow serpentine one, was the main phase where organic compounds were encountered (Fig. 7). Furthermore, only one pure component accounted for organic compounds in MCR-ALS analyses, hence suggesting only one single type of compounds homogeneously distributed in saponite. This pure component was characterized by a quite constant chemical signature whatever the analysed area (i.e. bending of aliphatic groups along with stretching of C=O carboxylic group and bending of N-H). The 1630 cm^−1^ vibrational band that was frequently observed in the organic-rich areas, both in S-FTIR raw spectra and after PCA, was then attributed without any confusion to the H-O-H bending of the secondary phyllosilicates^19,35,39^ and suggested again a close relationship between the organic compounds and this saponite phase in this sample. As largely acknowledged in the literature, this may overall suggest a potential role of saponite in the segregation and preservation of these organic compounds and likely in their abiotic formation, this mineral being well-known as a catalyst for organic synthesis^42–44^.

This close association of organic compounds with clays minerals such as saponite has already been described in carbonaceous meteorites that have undergone significant aqueous alteration processes^45–47^. Notably, S-FTIR analyses of meteoric grains revealed a strong correlation between hydrated phyllosilicates and organic compounds^6,7,48,49^. As proposed by ref.^45^, it might indicate a possible syngenetic link between these phyllosilicates, assumed to be formed in a geochemical process resembling serpentinization^50^, and organic compounds produced *in situ via* abiotic processes. All these studies suggest that clays minerals would have played a catalytic role in the polymerization of interstellar organic precursors and in the formation and preservation of prebiotic molecules with strong implications for the apparition of life on Earth. The results obtained in the present study confirm the intrinsic link between organic compounds and saponite but in serpentinized rocks from the terrestrial oceanic lithosphere. Although demonstrated for a young crust located close to the ridge, clay minerals and especially saponite derived from ultramafic rocks hydration are widespread on Earth. These serpentinization processes should have been also active on the primitive Earth where ultramafic minerals were abundant, giving credence to the hydrothermal theory for the origin of life^51,52^. Nonetheless, to improve our capability at deciphering the origin of the organic compounds encountered in such rocks, future work in chemometrics may also consider combining data cubes generated by complementary spectroscopic techniques (e.g. ToF-SIMS spectrometry, Raman spectroscopy, deep UV microscopy) which may help in individualizing organics, establish their signature and their link with the chemistry and mineralogy of their local environment.

Finally, this study opens new perspectives for the study of the under-explored reservoir of deep carbon on Earth. The nowadays-recognized capability of serpentinization to generate abiotic organic compounds and support an intraterrestrial life^29,53,54^ raise questions about the limits of the distribution of biotic *vs* abiotic organic carbon at depth and how it impacts the global carbon cycle. Combining microspectroscopic images and chemometrics on rocks from the oceanic crust, potentially harbouring organic compounds of both biological and abiotic origin will hence document to which extend the serpentinizing lithosphere might be seen as an important organic carbon factory on Earth.

## Material and Methods

### Sample preparation and mineralogy

The sample used in this study was a serpentinized mantle-derived peridotite collected at 170 m depth below seafloor (bsf) during the IODP drilling expeditions 304/305^34^ targeting the Atlantic Massif along the mid-Atlantic ridge (30°8′N–42°8′W). An inner sub-centimetric fragment was extracted from the IODP core by sawing under clean conditions using sterile deionized water and a saw blade treated with 5% sodium hypochlorite. It was followed by rinsing steps with sterile ultrapure water. Sub-sample dedicated to imaging techniques (i.e. optical microscopy, Scanning Electron Microscopy - SEM and S-FTIR microspectroscopy) was then prepared as a double-polished section. For this purpose, no resin or glue was used to avoid any organic contamination. The rock chip was manually gently thinned through successive polishing steps on both faces. The procedure was realized using silicon carbide polishing disks of decreasing size and sterile deionized water. Based on infrared attenuation coefficient, the sample thickness was estimated to be inferior to 100 μm and allowed FTIR measurements in transmission mode.

### Scanning Electron Microscopy

SEM observations were performed at the Service Commun de Microscopie Electronique à Balayage (UPMC, Paris, France) using a Zeiss SUPRA 55 VP Field Emission Scanning Electron Microscope. Samples were Au-coated. Images were collected using a backscattered electron detector (AsB) with accelerating voltage ranging from 10 kV to 15 kV at high current (up to 1 nA).

### S-FTIR microspectroscopy

S-FTIR hyperspectral imaging was performed at the French Synchrotron Radiation Facility SOLEIL (Saint-Aubin, France) on SMIS beamline^55^ by taking advantage of the brightness of the synchrotron source and of the confocal geometry of the microscope objective^56^. Up to 12 areas were mapped during the same experiment using a Nicplan microscope equipped with a ×32 Schwarzschild infinity corrected objective (N.A. = 0.65, Nicolet Reflachromat^™^) and coupled to a Magna FTIR spectrometer (Thermo Nicolet^™^). The microscope was equipped with a motorized sample stage (repeatability 1 μm) and a liquid nitrogen cooled mercury cadmium telluride (MCT-A – detector element size 250 μm) detector. The sample was deposited on a CaF_2_ window. Hyperspectral images were collected in transmission mode. The analytical conditions were the same for all data cubes’ acquisitions with a microscope aperture size of 5 × 5 μm^2^, a step size of 5 μm and a spectral resolution of 4 cm^−1^. Data cubes were collected using 64 accumulations per spectrum/pixel except for Area 1 (50 accumulations per spectrum).

Hyperspectral data cubes, in which pixels correspond to individual S-FTIR spectra have been unfolded in order to generate 2D matrices (Fig. 3a) and then analysed using the OMNIC^TM^ software (Thermo Fisher Scientific). A second derivative preprocessing was applied to spectra in order to remove baseline variations inherent to the analytical environment, the instrument itself, as well as the optical or physicochemical properties of natural heterogenous samples. Distribution maps of the aliphatic CH_2_ stretching peak intensity at 2848 cm^−1^ were generated in order to obtain a first estimate of the organic matter distribution^17^. We then selected 5 hyperspectral images (Areas 1, 2, 4, 6 and 9) presenting different mineralogical compositions (assessed optically using a petrographic microscope and chemically using SEM) and organic assemblages (Fig. 1). They provided a wide range of organic/mineral combinations that one can find in such a rock sample. They constituted a typical experimental dataset to decipher the micrometric associations between minerals and organics along with their nature in such a serpentinization environment. The 5 selected images (Fig. 1) represented areas of 80 × 105 μm^2^, 7 × 75 μm^2^, 100 × 85 μm^2^, 45 × 60 μm^2^ and 100 × 90 μm^2^ and were composed of 336, 225, 340, 108 and 360 spectra, respectively. Data were then processed for PCA/MCR-ALS analyses (see procedure hereafter).

### PCA/MCR-ALS analyses

Chemometric analyses were performed using Matlab^®^ software and the PLS toolbox (Eigenvector Research Inc.). Hyperspectral data cubes were analysed by PCA and MCR-ALS, individually (Fig. 3b) or as a multiset (Fig. 3c). Multiset mode was used to increase the variance of low-abundant components. Data cubes were combined by using the “concatenation” function of the Matlab^®^ software and PLS toolbox. In particular, the function “cat-img” has been used to concatenate dataset objects (dso).

In order to remove any non-informative signal variations or noise that can interfere with the chemical information carried by spectra, preprocessing was done on raw data for each individual data cube and combinations of them (Supplementary Fig. 6). In particular, because of the relatively large thickness of the sample, strong absorption occurred below 1500 cm^−1^ and above 3000 cm^−1^. Moreover, no peak was observed between 1800 and 2800 cm^−1^, whatever the analyzed area. We hence selected two spectral ranges, from 1430 to 1800 cm^−1^ and from 2820 to 2960 cm^−1^, which corresponded to main vibrational bands of both organic compounds and minerals with the best signal to noise ratio. We then applied a second derivative to raw data using the Stavitsky-Golay method (polynomial order: 2, filter width: 15) in order to eliminate baseline variations and highlight more absorption features. The selected spectral regions for individual images or multiset samples were then unit vector normalized and mean centered^57^.

For each single data cube or combination of them, a PCA model was developed using the PLS toolbox. Loadings and scores were recovered and the number of informative principal components (i.e. the rank of the chemical system) was noted. Due to the second derivative applied to spectra, the positions of peak maxima in loadings correspond to the absorbance band wavenumber in raw S-FTIR spectra. In order to identify the pure components of the system and to assess their spatial distribution, concatenated data cubes were then processed by MCR-ALS analyses (Fig. 3c).

### Data availability

The datasets generated and analysed in the present study are available from the corresponding author.

## Electronic supplementary material

Supplementary Information
