# Supplementary material for: Tracking hidden organic carbon in rocks using chemometrics and hyperspectral imaging
Source: Sci Rep. 2018 Feb 5;8:2396. doi: 10.1038/s41598-018-20890-4 (PMC5799262; doi:10.1038/s41598-018-20890-4)
Supplement: Supplementary file 1 — Supplementary Information [file 41598_2018_20890_MOESM1_ESM.pdf]

**Supplementary Material**

# Tracking hidden organic carbon in rocks using chemometrics and hyperspectral imaging

**Céline Pisapia[1,2,*], Frédéric Jamme[2], Ludovic Duponchel[3], Bénédicte Ménez[1]**

[1] IPGP, Sorbonne Paris Cité, Univ Paris Diderot, CNRS, 1 rue Jussieu, 75238 Paris Cedex 5, France
celine.pisapia@gmail.com, menez@ipgp.fr

[2] Synchrotron SOLEIL, Campus Paris-Saclay, 91192 Gif sur Yvette, France.
frederic.jamme@synchrotron-soleil.fr

[3] LASIR CNRS UMR 8516, Université de Lille, Sciences et Technologies, 59655 Villeneuve d'Ascq Cedex, France
ludovic.duponchel@univ-lille1.fr

[*] Corresponding author: PISAPIA Céline
- IPGP, 1 rue Jussieu, 75238 Paris Cedex 5, France
- Synchrotron SOLEIL, Campus Paris-Saclay, 91192 Gif-sur-Yvette, France
e-mail: celine.pisapia@gmail.com

**Supplementary Table 1.** Main S-FTIR vibrational bands observed in the different hyperspectral images and corresponding assigments[17,58,59]. It shows in particular the overlap in wavenumber of the bands characteristic of the different components expected in the sample. The presence of a S-FTIR vibrational band is noted with a cross for each Area.

| Wavenumber ($cm^{-1}$) | Assignment | Area 1 | Area 2 | Area 4 | Area 6 | Area 9 |
|---|---|---|---|---|---|---|
| 2850-2960 | C-H stretching of $CH_2$ and $CH_3$ | ✗ | ✗ | ✗ | - | ✗ |
| 1770-1780 | Si-O in olivine / carbonyl C=O stretching | - | ✗ | ✗ | ✗ | - |
| 1730 | Aromatic C=O stretching | ✗ | ✗ | ✗ | - | ✗ |
| 1600-1720 | Primary amines C=O and C-N stretching, N-H bending | ✗ | ✗ | ✗ | - | ✗ |
| 1610-1630 | H-O-H adsorbed in silicates | ✗ | ✗ | ✗ | - | ✗ |
| 1590-1600 | X-(O-H) bending in silicates (with X= Si, Mg, Fe) | ✗ | ✗ | ✗ | ✗ | ✗ |
| 1540-1570 | X-(O-H) bending in silicates (with X= Si, Mg, Fe) | ✗ | ✗ | ✗ | ✗ | ✗ |
| 1550-1650 | Primary amine $NH_2$ scissoring, aromatic C=C stretching | ✗ | ✗ | ✗ | - | ✗ |
| 1500-1570 | Secondary amines C-N stretching and N-H bending | ✗ | ✗ | ✗ | - | ✗ |
| 1450-1470 | N-H bending of $NH_2$, C-H bending of $CH_2$ and $CH_3$ | ✗ | ✗ | ✗ | - | ✗ |
| 1400-1500 | X-(O-H) bending in silicates (with X= Si, Mg, Fe) | ✗ | ✗ | ✗ | ✗ | ✗ |

**Supplementary Table 2.** Synthetic table of the main results obtained after Principal Component Analysis performed on individual data cubes and multiset combinations of them. The number (Nb) of significant principal components (PC) proposed by Matlab® software is indicated and the percentage of total variance (% variance) expressed by each PC is detailed. Phase contributions for each principal component are proposed for the positive (>0) and the negative (<0) panels, based on scores and loadings interpretation with Ol: olivine, w-Srp: white serpentine, y-Srp: yellow serpentine, Sap: saponite, orga: organic compounds, vw-Srp: white serpentine from late vein and *n.a.*: not assigned.

| **Area(s)** | | | **2** | **2+4** | **2+1** | **1+2+4+6+9** |
|---|---|---|---|---|---|---|
| Nb of significant PC | | | 3 | 4 | 5 | 5 |
| PC1 | % variance | | 42.5 | 48.9 | 46.5 | 29.1 |
| | Phases contribution | >0 | Ol, orga | Ol, orga | orga | orga |
| | | <0 | w-Srp | w-Srp | w-Srp, Ol | w-Srp, Ol |
| PC2 | % variance | | 14.2 | 12.2 | 21.6 | 19.8 |
| | Phases contribution | >0 | orga | orga | Ol | Ol |
| | | <0 | Ol, w-Srp | Ol, w-Srp | w-Srp | w-Srp |
| PC3 | % variance | | 12.1 | 7.3 | 5.7 | 12.0 |
| | Phases contribution | >0 | Sap | Sap | Sap | Sap |
| | | <0 | w-Srp, Ol | orga | Ol, w-Srp, orga | Ol, orga |
| PC4 | % variance | | 2.6 | 2.8 | 5.3 | 4.2 |
| | Phases contribution | >0 | *n.a.* | *n.a.* | orga | Sap, orga, Ol, w-Srp |
| | | <0 | *n.a.* | *n.a.* | *n.a.* | |
| PC5 | % variance | | - | - | 2.9 | 3.6 |
| | Phases contribution | >0 | - | - | *n.a.* | *n.a.* |
| | | <0 | - | - | *n.a.* | vw-Srp |

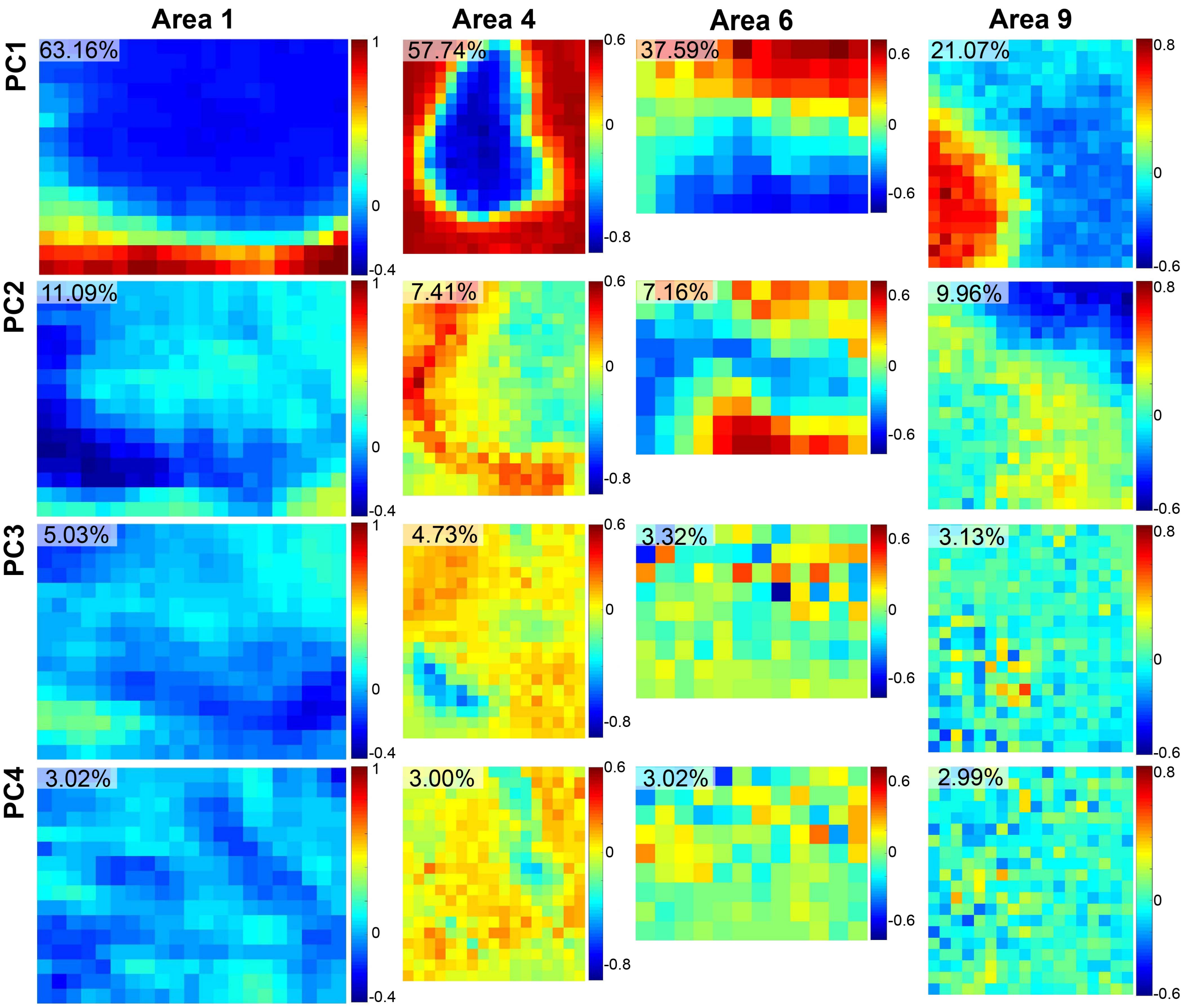


**Supplementary Figure 1.** PCA results obtained on individual images Areas 1, 4, 6 and 9 following preprocessing procedure (i.e. wavenumber range selection, 2[d] derivative, normalization and mean centering). Scores of each principal component (PC) are presented with the associated percentage of total variance explained.

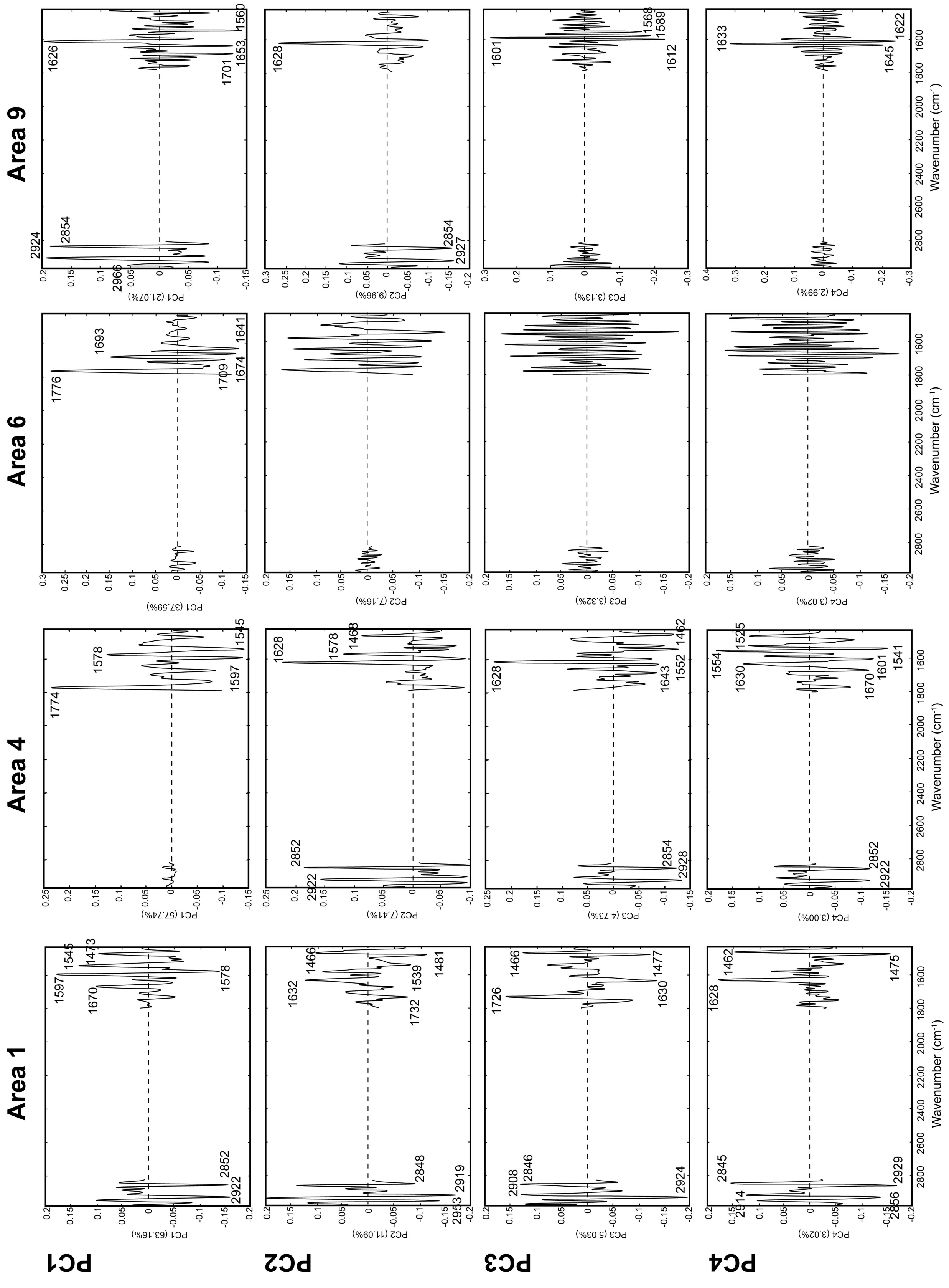


**Supplementary Figure 2.** PCA results obtained on individual images Areas 1, 4, 6 and 9 following preprocessing procedure (i.e. wavenumber range selection, 2$^{d}$ derivative, normalization and mean centering). Loadings of each principal component (PC) are presented with the associated percentage of total variance explained.

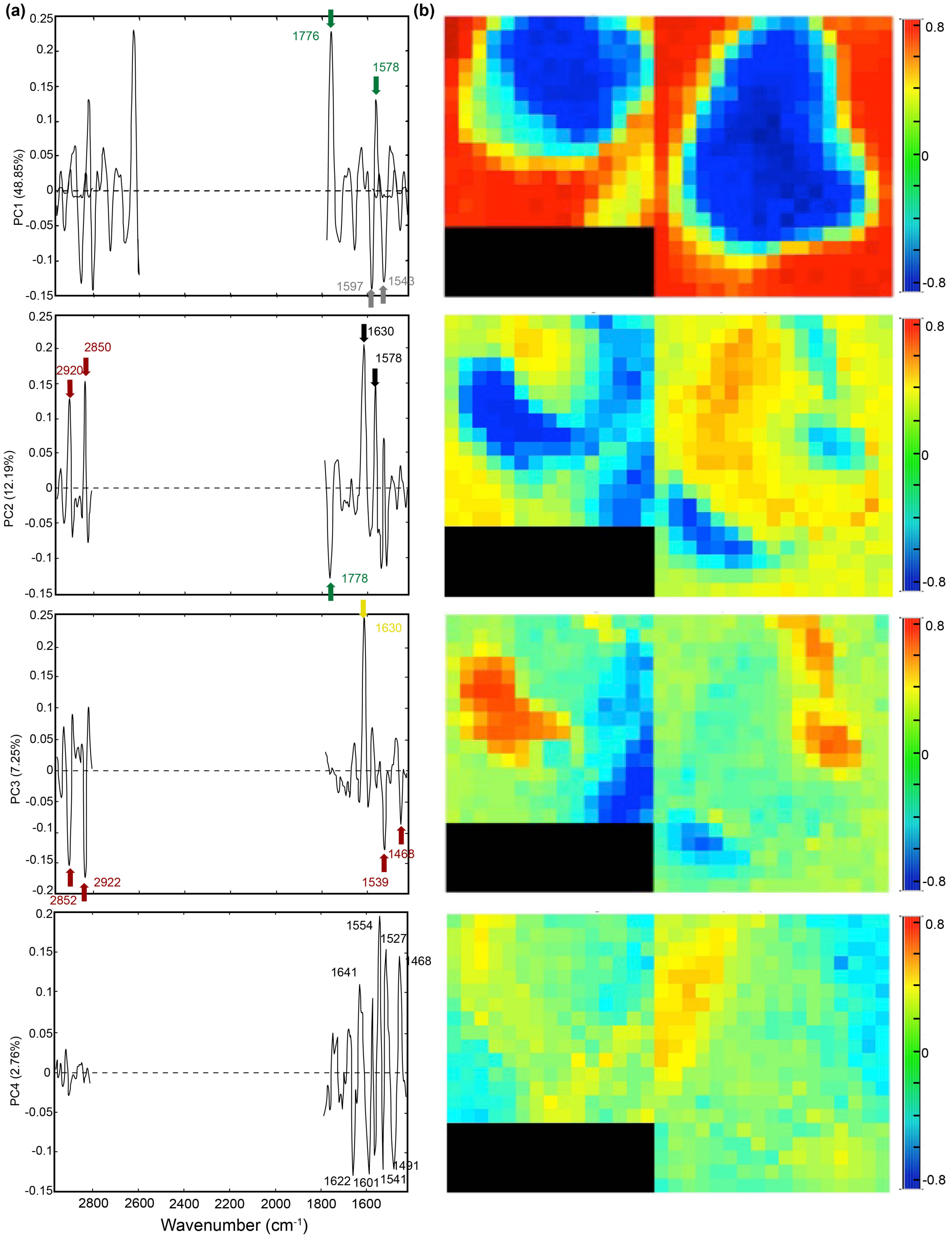


**Supplementary Figure 3.** PCA results obtained when combining two S-FTIR data cubes of similar composition (Areas 2 and 4) following the preprocessing procedure (i.e. wavenumber range selection, 2$^{d}$ derivative, normalization and mean centering). Loadings **(a)** and scores **(b)** are shown for principal components (PC) 1 to 4. The percentage of total variance explained by the principal component is given along the *y*-axis. Main peaks on loadings are highlighted and were assigned to organic compounds (red arrows), saponite (yellow arrows), olivine (green arrows) and white serpentine (grey arrows). Peaks values noted in black were not assigned.

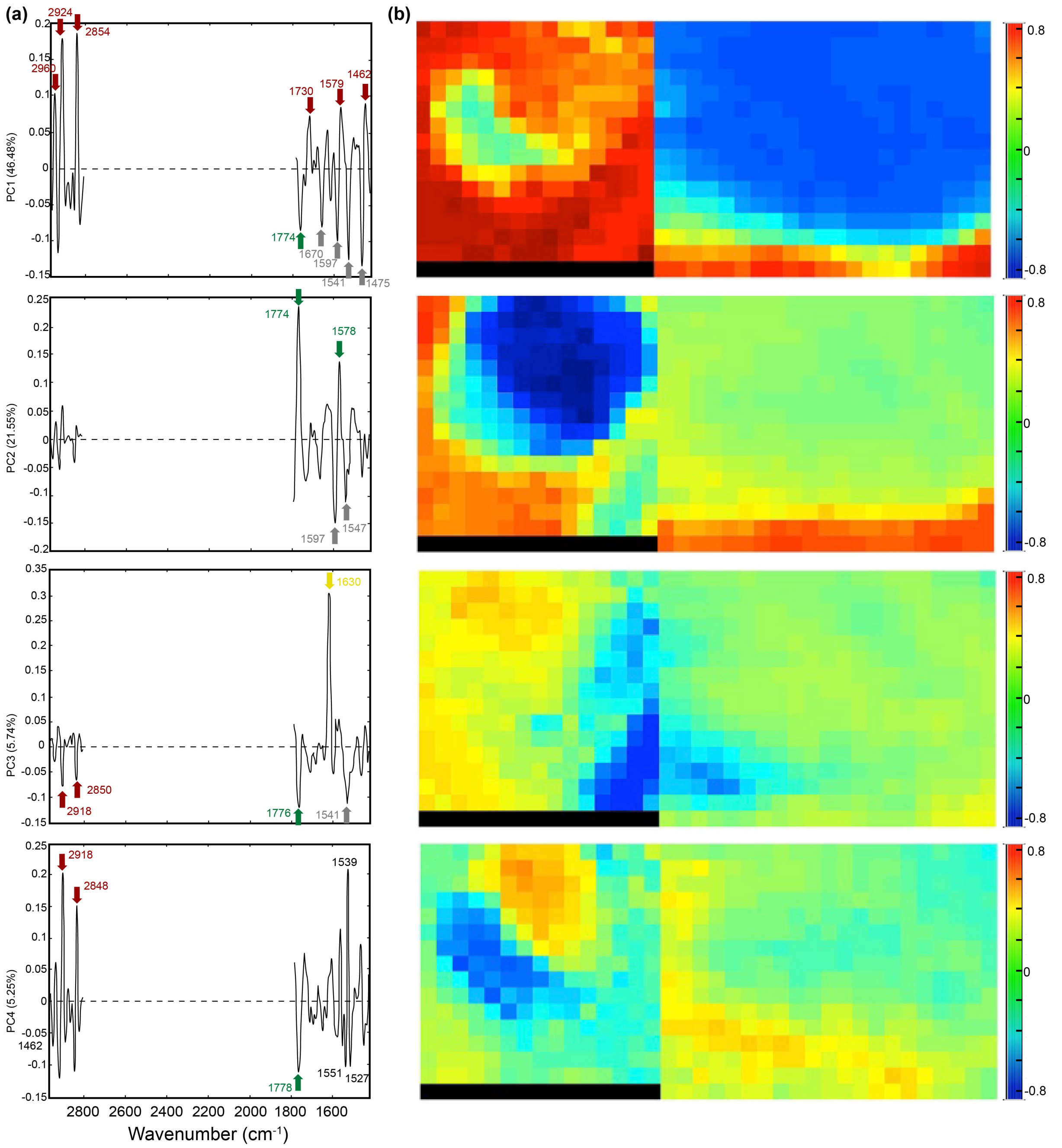


**Supplementary Figure 4.** PCA results obtained by combining 2 S-FTIR data cubes of different composition (Areas 1 and 2) following the preprocessing procedure (i.e. wavenumber range selection, 2$^{d}$ derivative, normalization and mean centering). Loadings **(a)** and scores **(b)** are shown for principal components (PC) 1 to 4. The percentage of total variance explained by the principal component is given along the *y*-axis. Main peaks on loadings are highlighted and were assigned to organic compounds (red arrows), saponite (yellow arrows), olivine (green arrows) and white serpentine (grey arrows). Peaks values noted in black were not assigned.

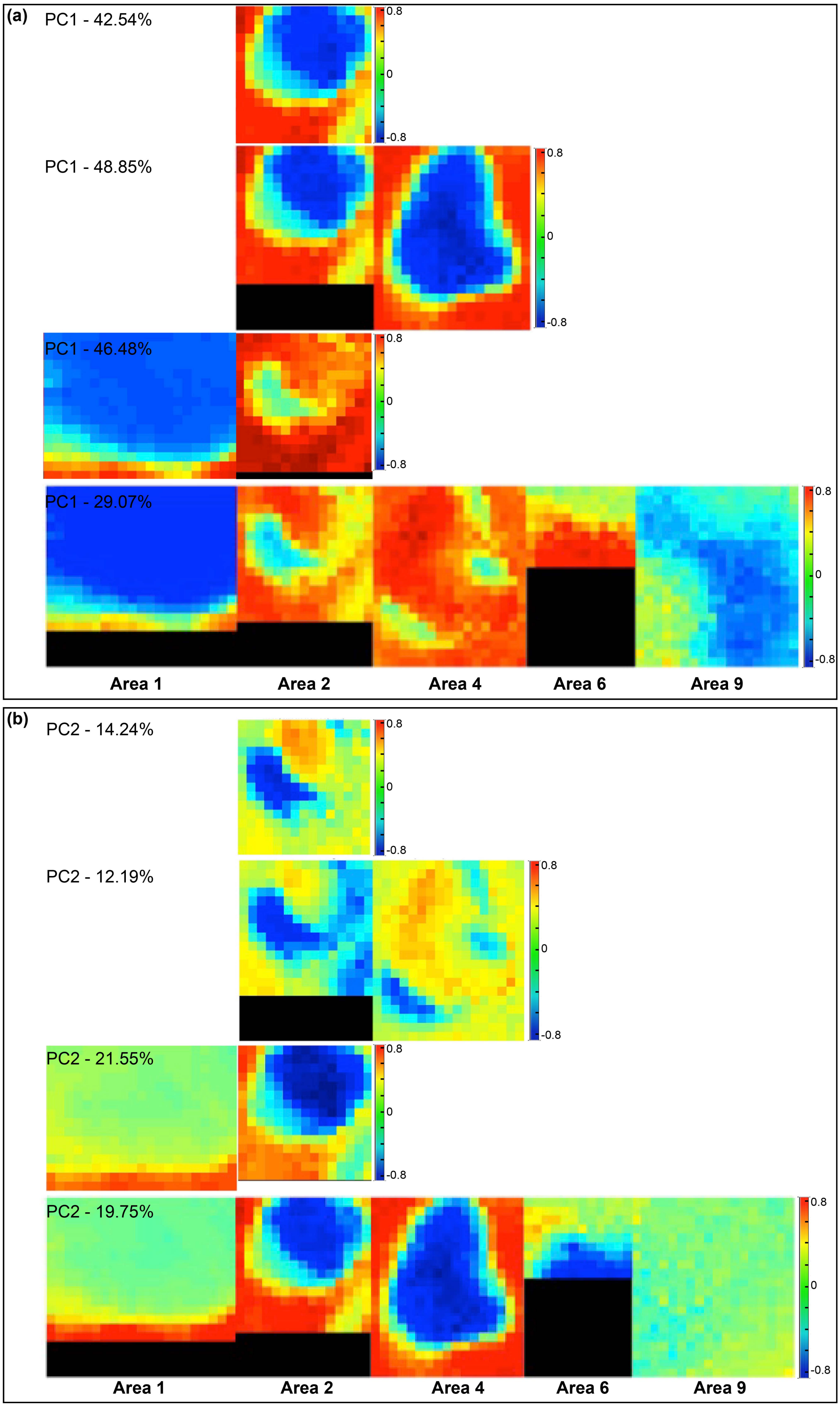


**Supplementary Figure 5.** Influence of multiset analyses on the scores of the first **(a)** and second **(b)** principal component (PC) obtained on the single image Area 2 (Fig. 4) or after combination with a data cube of similar composition (Area 4; Supplementary Fig. 3) or of different composition (Area 1; Supplementary Fig. 4) or combination of all data cubes (Areas 1, 4, 6 and 9; Fig. 5). For all configurations, the percentage of total variance explained by the principal component is given.

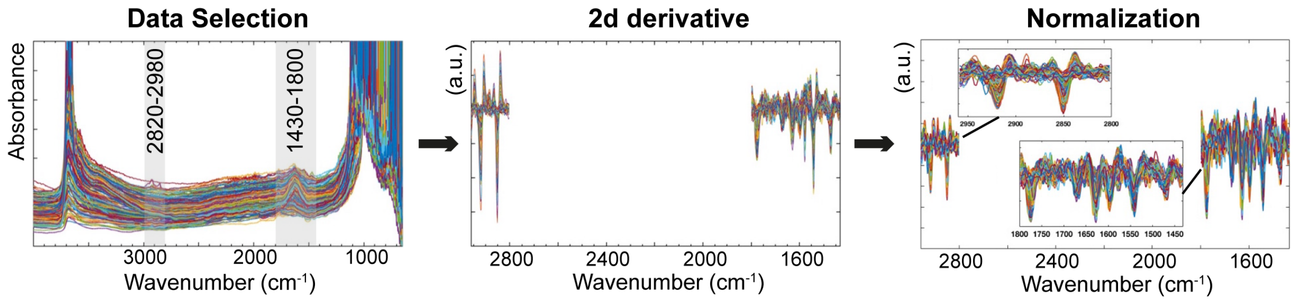


**Supplementary Figure 6.** Preprocessing applied on individual or on concatenated S-FTIR hyperspectral data cubes. Two wavenumber ranges (1430-1800 $cm^{-1}$ and 2820-2960 $cm^{-1}$) were first selected. Second derivative and normalization processing were then applied. Finally, data were unit vector normalized and mean centered, and then analysed using PCA and MCR-ALS.
